# Supplementary material for: Genetic evidence that Chinese chestnut cultivars in Japan are derived from two divergent genetic structures that originated in China
Source: PLoS One. 2020 Jul 1;15(7):e0235354. doi: 10.1371/journal.pone.0235354 (PMC7329096; doi:10.1371/journal.pone.0235354)
Supplement: S1 Table — (PDF) [file pone.0235354.s001.pdf]

S1 Table. Names, accession numbers, and information for the 231 cultivars used in this study.

Genotype	Cultivar	Orignal name	Institute (Accession no.)	Origin	Code (group number)	PEA41	PRD52	PRD2	PRB9	PRA76	PRD67	PEA52	PRD84	CmSI0931	PRA35	PRA42	PRA83	PRF84	PRD53	PEB32	PRA67	PEA74	EMCs2	PRA34	PRF54	PEB47	CmSI0513	PEA86	PEB55	PRD55	PRG79	CmSI0771	CmSI0433	CmSI0668	CmSI0045	PRD83	Cmcs1 (KY951992:82981)	Cmcs2 (KY951992:39981)	Cmcs3 (KY951992:69174)	Cmcs5 (KY951992:125578)	Cmcs7 (KY951992:130088)	Chloroplast haplotype
1	Arima	有磨	Institue of Fruit Tree and Tea Science, NARO (113832)	Kanto (Japan)	J_KA (1)	104/106	145/151	131/143	108/120	257/259	113/115	81/106	142/142	299/302	184/208	302/304	251/251	141/141	146/146	163/163	260/264	150/152	166/168	183/183	132/142	152/162	183/193	145/151	163/175	152/160	186/206	276/280	152/164	232/232	219/227	132/138	99	143	162	151	210	HAP1
2	ChuutanA	中丹A	Institue of Fruit Tree and Tea Science, NARO (113837)	Kanto (Japan)	J_KA (1)	104/104	141/145	143/143	108/120	259/263	113/115	93/93	134/150	302/302	192/206	300/302	251/253	141/145	142/146	163/165	260/266	150/152	168/168	169/183	130/146	156/162	183/185	151/151	165/171	138/156	180/214	280/282	162/164	222/224	209/219	132/146	99	143	162	151	210	HAP1
3	Gosha	後社	Institue of Fruit Tree and Tea Science, NARO (113851)	Kanto (Japan)	J_KA (1)	104/104	151/151	133/147	120/120	248/257	111/111	93/93	142/150	296/302	178/184	302/304	251/251	147/147	146/146	163/163	258/260	146/150	168/168	169/169	140/140	156/162	185/189	145/151	163/163	138/156	220/220	272/280	164/166	232/232	217/219	148/148	99	143	162	151	210	HAP1
4	Hassaku	八朔	Institue of Fruit Tree and Tea Science, NARO (113853)	Kanto (Japan)	J_KA (1)	104/108	145/151	143/143	120/120	248/273	111/111	93/93	134/136	302/302	184/184	302/304	243/251	145/147	146/146	163/163	260/260	150/150	160/168	171/183	132/132	162/164	185/187	111/151	163/175	142/142	182/184	280/280	166/166	228/228	209/225	132/132	99	143	162	151	210	HAP1
5	Moriwase	森早生	Institue of Fruit Tree and Tea Science, NARO (176784)	Kanto (Japan)	J_KA (1)	106/108	143/143	133/141	108/120	259/259	103/113	93/93	136/140	302/302	184/188	302/303	245/251	141/145	144/146	163/165	254/260	146/150	160/160	183/185	140/142	162/164	185/187	151/151	163/173	144/144	180/210	282/288	162/164	224/228	217/225	132/156	99	143	162	151	210	HAP1
6	Nakatetanba	中生丹波	Institue of Fruit Tree and Tea Science, NARO (176785)	Kanto (Japan)	J_KA (1)	104/108	145/145	143/143	108/108	257/263	113/113	93/106	134/150	302/305	184/206	302/304	245/251	145/147	142/144	165/165	254/266	146/152	166/168	183/183	146/146	156/162	183/187	143/151	171/173	140/156	214/228	282/290	164/166	224/224	209/227	146/148	99	143	162	151	210	HAP1
7	Odai	小台	Institue of Fruit Tree and Tea Science, NARO (113881)	Kanto (Japan)	J_KA (1)	102/104	143/151	133/143	104/120	257/257	99/113	93/93	134/140	302/302	192/208	288/288	245/253	141/147	146/146	160/165	260/260	146/146	166/168	173/173	132/144	152/156	185/197	145/151	163/175	152/156	188/218	282/288	164/164	228/232	217/227	132/138	99	143	162	151	210	HAP1
8	Osaya	筬屋	Institue of Fruit Tree and Tea Science, NARO (113884)	Kanto (Japan)	J_KA (1)	104/106	145/151	143/147	104/120	257/259	113/119	99/105	142/154	302/302	178/198	304/310	245/245	141/147	142/142	163/165	260/270	150/150	168/168	171/183	132/142	162/164	187/193	151/151	173/179	142/152	188/226	276/282	152/166	224/228	219/225	132/146	99	143	162	151	210	HAP1
9	Senri	千里	Institue of Fruit Tree and Tea Science, NARO (113886)	Kanto (Japan)	J_KA (1)	102/102	143/151	133/143	104/120	248/273	99/119	93/105	140/150	302/302	184/190	302/304	245/251	147/155	148/148	163/163	258/264	146/146	160/168	171/183	129/146	156/162	185/189	145/151	163/163	138/143	210/218	280/290	156/164	232/232	209/225	146/146	99	143	162	151	210	HAP1
10	Shichifukuwase	七福早生	Institue of Fruit Tree and Tea Science, NARO (113891)	Kanto (Japan)	J_KA (1)	104/106	141/143	133/143	104/120	248/248	113/115	93/106	134/150	302/302	184/184	288/304	251/251	139/141	142/146	163/163	264/264	146/150	160/168	171/183	132/148	162/162	185/185	145/151	163/187	138/160	206/220	280/290	156/164	232/232	223/227	132/132	99	143	162	151	210	HAP1
11	Taishouwase	大正早生	Institue of Fruit Tree and Tea Science, NARO (113897)	Kanto (Japan)	J_KA (1)	104/108	143/151	143/143	108/120	257/257	113/113	93/93	140/140	293/302	184/188	302/304	245/251	141/145	144/144	163/163	264/264	150/150	160/168	169/183	140/142	152/162	185/193	145/151	163/173	140/152	180/206	272/282	162/164	232/232	217/225	132/144	99	143	162	151	210	HAP1
12	Toyotamawase	豊多摩早生	Institue of Fruit Tree and Tea Science, NARO (113907)	Kanto (Japan)	J_KA (1)	104/106	143/145	141/147	108/120	259/273	111/113	93/93	134/136	296/302	178/184	280/302	251/251	141/145	142/146	163/163	260/260	150/150	160/166	171/183	132/142	156/164	187/193	125/151	173/175	138/144	180/190	288/290	164/164	228/232	209/217	132/132	99	143	162	151	210	HAP1
13	Tsunehisa	常久	Institue of Fruit Tree and Tea Science, NARO (113910)	Kanto (Japan)	J_KA (1)	104/108	145/145	143/147	120/120	248/248	109/113	93/93	134/134	296/302	184/206	304/316	245/251	141/141	148/148	163/163	260/270	146/150	160/169	171/183	142/142	156/162	185/189	125/145	163/173	144/160	188/214	276/280	164/166	228/232	227/227	132/148	99	143	162	151	210	HAP1
14	Yamatowase	大和早生	Institue of Fruit Tree and Tea Science, NARO (113916)	Kanto (Japan)	J_KA (1)	104/106	143/145	143/143	108/120	248/257	113/115	106/106	142/150	302/302	184/208	302/304	251/251	141/145	142/146	163/165	254/260	146/152	160/166	171/183	146/148	152/156	183/187	143/151	163/173	156/160	206/214	280/282	164/164	224/232	209/227	132/146	99	143	162	151	210	HAP1
15	Choubei	長兵衛	Institue of Fruit Tree and Tea Science, NARO (113838)	Tanba (Japan)	J_TA (2)	102/102	141/145	143/147	104/104	248/259	99/99	93/106	134/138	302/305	184/184	288/302	245/245	141/141	142/146	160/163	260/266	146/150	166/166	171/171	140/146	160/164	183/200	125/125	163/163	144/156	182/226	280/286	164/166	222/232	209/217	132/152	99	143	162	151	210	HAP1
16	Choukouji	長光寺	Institue of Fruit Tree and Tea Science, NARO (113839)	Tanba (Japan)	J_TA (2)	106/108	143/151	133/147	108/120	257/259	103/111	93/93	142/142	302/305	184/188	302/318	253/253	143/147	142/148	163/165	256/266	150/150	166/169	169/169	134/140	152/162	185/197	151/151	163/175	138/152	180/188	284/284	162/162	232/232	227/229	132/140	99	143	162	151	210	HAP1
17	Daihachi	大八	Institue of Fruit Tree and Tea Science, NARO (113841)	Tanba (Japan)	J_TA (2)	102/104	145/151	143/143	104/104	248/263	99/113	93/93	140/142	302/302	184/192	288/288	245/251	141/141	146/148	160/163	260/260	146/150	160/166	171/173	134/146	160/160	187/200	125/125	173/179	144/156	180/224	272/280	164/164	232/232	219/227	132/132	99	143	162	151	210	HAP1
[illegible]	[illegible]	[illegible]	[illegible]	[illegible]	[illegible]	[illegible]	[illegible]	[illegible]	[illegible]	[illegible]	[illegible]	[illegible]	[illegible]	[illegible]	[illegible]	[illegible]	[illegible]	[illegible]	[illegible]	[illegible]	[illegible]	[illegible]	[illegible]	[illegible]	[illegible]	[illegible]	[illegible]	[illegible]	[illegible]	[illegible]	[illegible]	[illegible]	[illegible]	[illegible]	[illegible]	[illegible]	[illegible]	[illegible]	[illegible]	[illegible]	[illegible]	[illegible]
