# Supplementary figures and images for: Genetic evidence that Chinese chestnut cultivars in Japan are derived from two divergent genetic structures that originated in China
Source: PLoS One. 2020 Jul 1;15(7):e0235354. doi: 10.1371/journal.pone.0235354 (PMC7329096; doi:10.1371/journal.pone.0235354)

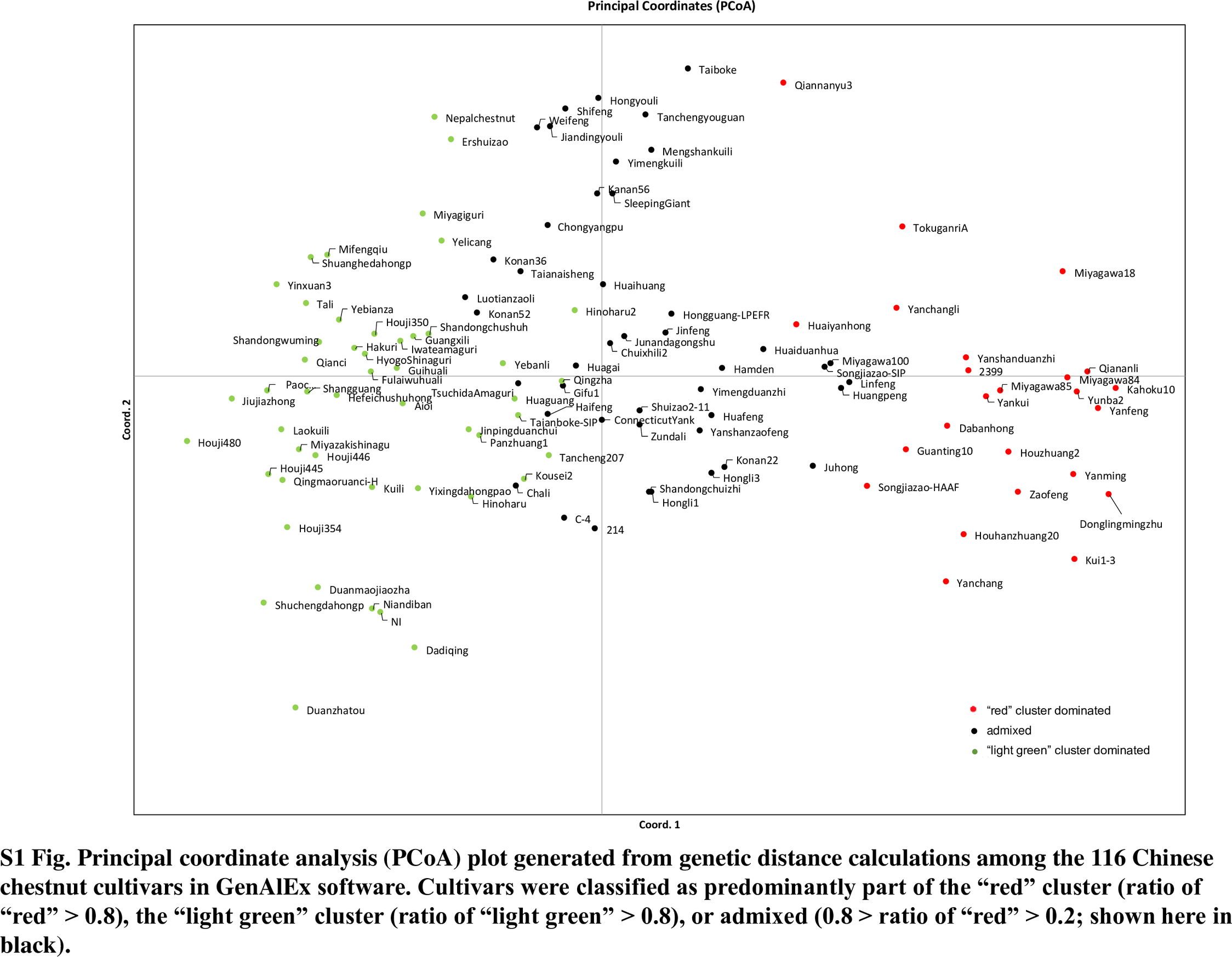

Supplement: S1 Fig — Cultivars were classified as predominantly part of the “red” cluster (ratio of “red” > 0.8), the “light green” cluster (ratio of “light green” > 0.8), or admixed (0.8 > ratio of “red” > 0.2; shown here in black). (TIF) [file pone.0235354.s004.tif]
