# Supplementary material for: Genetic evidence that Chinese chestnut cultivars in Japan are derived from two divergent genetic structures that originated in China
Source: PLoS One. 2020 Jul 1;15(7):e0235354. doi: 10.1371/journal.pone.0235354 (PMC7329096; doi:10.1371/journal.pone.0235354)
Supplement: Attachment — Submitted filename: 200225Response to Reviewers.docx [file pone.0235354.s005.docx]

**Response to the Associate Editor**

We thank the Editor and both Reviewers very much for taking time to review our manuscript and for your comments. We have revised our manuscript accordingly.

**Responses to the comments from Reviewer 1**

**Comment**

1. Figure 1: The group C_SJ is clustered (100% green cluster) with the Chinese accessions. It’s not clear if all the Chinese chestnuts selected in Japan were originated from the first introduction of seeds. Could some accession haven been selected from successive generation of natural hybridisation with Japanese genepool?

**Response**

There were some cultivars in C_SJ that had originated from the first introduction of seeds into Japan: for example, ‘Houji’ and ‘Miyagawa’ had records of breeding history indicating that they were directly selected from seeds introduced from China into Japan. But others would have been selected from successive generations of the introduced genotypes, as this reviewer suggested. To clarify this point, we revised several sentences in the Discussion (Line 314, 327-329)

.

**Comment**

2. Lin 357-366: T The argument presented to support the origin of C_SJ group of accessions is not strong. I suggest authors to present neighbour-joining (NJ) and principal component analysis (PCA) of nSSR genotypes to further support their hypothesis.

**Response**

We performed principal coordinate analysis (PCoA; S1 Fig) and describe the results in the indicated paragraph (Line 419-422).

**Comment**

3. Line 150: Authors should describe the steps for constructing chloroplast haplotypes. Some comments on the extent of linkage disequilibrium would be helpful in order to check the integrity.

**Response**

According to this suggestion, we now describe in the Materials and Methods how we classified the haplotypes (Line 159-162). We also added the positions of the 5 cpSSRs in the complete *Castanea mollissima* chloroplast genome (KY951992) to S1 Table.

**Comment**

4. Could a relatively lower chloroplast diversity (hence a simpler genetic structure) be a result of only 5 cpSSR used in this study?

**Response**

Only two haplotypes (Chen et al., 2009) and four haplotypes (this study) were identified from Chinese chestnut cultivar collections because small numbers of cpSSRs (4 and 5, respectively) developed by Sebastiani et al. (2004) were used in those studies. Liu et al. (2013) also used a small number of cpSSRs (7) to clarify the genetic relationship between wild populations, in which they identified 38 chloroplast haplotypes. It is reasonable to compare the results of our study and of those previous studies because the numbers and types of markers were quite similar. On the other hand, it is possible that more chloroplast haplotypes would be found if we sequenced the whole chloroplast genomes of several cultivars and identified SNPs. We revised the text to clarify this point (Line 382-389).

**Comment**

5. It would be useful if authors could provide information on the distribution of 31 SSRs across different chromosomes. Perhaps using 31 SSRs would only detect genetic diversity of limited regions of the whole genome, and could lead to biased inferences about population genetics. Perhaps authors could discuss this point.

**Response**

The information on distribution of the 31 SSRs is provided in S2 Table and shows that the markers were mapped on different chromosome regions. The number of SSRs in this study was larger than that in most population genetic studies. Since we applied reliable SSRs sampled from among more than 500 developed in several previous studies, we have confidence in our results. Using a large number of SNPs from next-generation sequencing (NGS) might increase the amount of information; on the other hand, genotyping using NGS is not always precise because genomes contain much duplication.

**Responses to Reviewer 2**

**Comment**

1. What is the purpose (or significance) to reveal the origin of Chinese chestnut cultivars introduced into Japan? If authors would like to understand the origin of Japanese chestnut cultivars in relation to Chinese chestnut, materials used in this study were not enough. More Chinese chestnut cultivars in China should be involved.

**Response**

We revised several sentences in the Introduction to clarify the objective of this study (Line 52-60). Interspecific hybridization has been done by chestnut breeders all over the world to introduce disease resistance genes, an easy-peeling gene, and QTLs associated with nut quality into their breeding materials. Therefore, classification of cultivars in Japanese, Chinese, and their hybrids is quite important for breeders.

At the present time, materials from China are difficult to introduce into other countries because the Nagoya protocol (2017) prohibits exchange of cultivars. We examined 84 Chinese chestnut cultivars from 4 different provinces in China, that are moderate numbers and quite valuable for the first report of Chinese chestnut cultivar classification by SSRs. In addition, Chinese chestnut cultivars already in Japan have been preserved at the NARO (National Agriculture and Food Organization) Genebank (www.gene.affrc.go.jp) and can be readily used for research and breeding purposes all over the world. Thus, it is useful to clarify the genetic structure of these cultivars.

**Comment**

2. Nuclear SSR markers were applied to reveal the genetic structure of Chinese and Japanese cultivars. But the resolution of SSR marker seems too high to apply for different species i.e., Castanea mollissima and C. crenata. Therefore only three clusters such as Chinese cultivar cluster, Japanese cultivar cluster and hybrid cluster were obtained. Is it possible to use other molecular markers such as the sequence of single nuclear gene or gene encoding chloroplast genome?

**Response**

As the reviewer suggests, applying nuclear SSRs is not the best way to determine the genetic distance between different species. In this study, we applied SSRs only to identify synonym groups (first step) and to classify Japanese, Chinese, and Japanese–Chinese hybrid cultivars and determine the parentage of the hybrids (second step) not to determine genetic distances between cultivars of different species. The high level of SSR polymorphism was no problem for these analyses. We then performed additional classification within the group of Chinese chestnut cultivars (third step), which revealed two divergent genetic structures, i.e., Hebei origin and Jiangsu or Anhui origin. The values of Δ(*K*) were much higher at *K* = 2 than at *K* = 3 to *K* = 8 in Bayesian structure analysis, suggesting that there would be no more than 2 clusters in the Chinese cultivar collection.

**Comment**

3. Authors say this research is useful for chestnut breeding program. But it is too general. Is there any information concerning agricultural traits for used cultivars? For example, is there any excellent or desirable trait inherited from putative parent to offspring?

**Response**

To provide an example, we added the story of the hybrid cultivar Riheiguri, which is one of the major cultivars in Japan and has good nut quality and moderate peelability derived from Chinese chestnut (Line 362-375).

**Comment**

This manuscript seems suitable to submit to more specific journal focused on the plant breeding or woody plants genetics.

**Response**

Thank you for your comment. We considered our manuscript appropriate for submission because we have seen a number of similar population genetic studies reported in *PLoS One*, and we have attempted to follow the journal’s policies and guidelines.
