# Supplementary material for: Genetic evidence that Chinese chestnut cultivars in Japan are derived from two divergent genetic structures that originated in China
Source: PLoS One. 2020 Jul 1;15(7):e0235354. doi: 10.1371/journal.pone.0235354 (PMC7329096; doi:10.1371/journal.pone.0235354)
Supplement: Attachment — Submitted filename: 200426Response to Reviewers.docx [file pone.0235354.s006.docx]

**Responses to the comments from the Editor**

We thank the Editor and both Reviewers very much for taking time to review our manuscript and for your comments. We have revised our manuscript accordingly.

**Comment**

I'd like to ask you to consider to add data of wild accessions and/or agronomic traits as suggested by the Reviewer 2.

**Response**

In response to this suggestion, we added the nut harvest date and nut weight of Japanese chestnut cultivars, Japanese–Chinese hybrid cultivars, and Chinese chestnut cultivars selected in Japan in S3 Table. We also revised the text to describe the differences in average weight between the three species groups.

In this study, we focused on genetic structure analysis of Japanese, Chinese, and Japanese–Chinese hybrid chestnut cultivars. The genetic relationships between cultivars and wild accessions are another story and should be analyzed using only pure Japanese chestnut. Because of the complexity of including both cultivars and wild accessions of both Japanese and Chinese chestnut in a single study, such species classification and domestication studies are beyond the scope of this report. The genetic structure information on the cultivars described in this report will be useful for further studies related to the domestication process.

**Response to the comment from Reviewer 1**

**Comment**

However, I could not find Supporting Information S1 Fig.

**Response**

We added S1 Fig to the revised manuscript.

**Responses to the comments from Reviewer 3**

**Comment**

However, as for this level of journal (PLOSone), I feel wild accessions should be included or trait data. Particularly, author estimated the offspring and how many crosses had been done. Then, I am very interested in how their agronomical traits.

**Response**

We do not agree with Reviewer 3 on this point. We found a number of studies including only cultivars and accessions (i.e., not including wild accessions or phenotypic data) published in *PLoS ONE* within the past three years. Here are some examples:

Arnau, G., Bhattacharjee, R., Sheela, M. N., Chair, H., Malapa, R., Vincent Lebot, A. K., ... & Pavis, C. (2017). Understanding the genetic diversity and population structure of yam (*Dioscorea alata* L.) using microsatellite markers. *PLoS ONE*, *12*(3), e0174150.

Manechini, J. R. V., da Costa, J. B., Pereira, B. T., Carlini-Garcia, L. A., Xavier, M. A., de Andrade Landell, M. G., & Pinto, L. R. (2018). Unraveling the genetic structure of Brazilian commercial sugarcane cultivars through microsatellite markers. *PLoS ONE*, *13*(4), e0195623.

Urrestarazu, J., Errea, P., Miranda, C., Santesteban, L. G., & Pina, A. (2018). Genetic diversity of Spanish *Prunus domestica* L. germplasm reveals a complex genetic structure underlying. *PLoS ONE*, *13*(4), e0195591.

Bernard, A., Barreneche, T., Lheureux, F., & Dirlewanger, E. (2018). Analysis of genetic diversity and structure in a worldwide walnut (*Juglans regia* L.) germplasm using SSR markers. *PLoS ONE*, *13*(11), e0208021.

Zhu, S., Zhang, X., Liu, Q., Luo, T., Tang, Z., & Zhou, Y. (2018). The genetic diversity and relationships of cauliflower (*Brassica oleracea* var. *botrytis*) inbred lines assessed by using SSR markers. *PLoS ONE*, *13*(12), e0208551.

Atnaf, M., Yao, N., Martina, K., Dagne, K., Wegary, D., & Tesfaye, K. (2017). Molecular genetic diversity and population structure of Ethiopian white lupin landraces: Implications for breeding and conservation. *PLoS ONE*, *12*(11), e0188696.

Nevertheless, we added phenotypic data for nut harvest date and nut weight of cultivars preserved at the NARO Genebank in Japan. Such data were not available for the Chinese chestnut cultivars from China, for which we only had DNA samples.

**Comment**

And also in synonymous strains whether they share same kinds of traits.

**Response**

All of the synonym groups we identified were Chinese chestnut cultivars from China. As described above, only DNA samples were available for these cultivars, so we could not perform phenotypic analysis. However, in a previous study (Nishio et al. 2011), cultivars that had identical genotypes showed similar phenotypic traits.

Nishio S, Yamamoto T, Terakami S, Sawamura Y, Takada N, Saito T. Genetic diversity of Japanese chestnut cultivars assessed by SSR markers. *Breeding Sci.* 2011;61(2):109-20. doi: Doi 10.1270/Jsbbs.61.109.

**Comment**

Kinship is another issue. Even small number of SSRs, phylogenetic relationship is important data to consider breeding program. In addition, further research like GWAS, the preliminary data are required to conduct further research. In this meaning, authors can add these data and supposed to do. By the other hand, if not, authors are recommended to try other journals.

**Response**

The results obtained using phylogenetic trees sometimes fluctuate depending on the parameters selected or the method of calculation. Instead of showing phylogenetic trees, we present the results of principal coordinate analysis (PCoA; S1 Fig) to clarify the genetic relationships between cultivars. The Bayesian structure and PCoA results will be useful for both chestnut breeding programs and further genetic studies including GWAS and population genetics.

**Comment**

Obiwase (#ID is required)

Some parts of cultivars are referred ID #, however, some are not. It is hard to follow the cultivar in Figure of STRUCTURE analysis to know how they are in data.

**Response**

In response to this suggestion, we now include both cultivar names and ID numbers in the text.

**Comment**

“The sixth" is hard to get the meaning. One the sixth cultivars you mentioned?

Then, Just "Ikaba" is enough. If there are other Ikaba cultivars, please show ID#.

**Response**

In response to this suggestion, we combined and revised the sentences mentioned here.
