# Supplementary material for: Genetic evidence that Chinese chestnut cultivars in Japan are derived from two divergent genetic structures that originated in China
Source: PLoS One. 2020 Jul 1;15(7):e0235354. doi: 10.1371/journal.pone.0235354 (PMC7329096; doi:10.1371/journal.pone.0235354)
Supplement: Attachment — Submitted filename: 200609Response to Reviewers.docx [file pone.0235354.s007.docx]

**Responses to the comments from the Editor**

We thank the Editor and both Reviewers very much for taking time to review our manuscript and for your comments. We have revised our manuscript accordingly. During the revision, we found that there was a set of duplicated genotypes in the C_IO. Therefore, we revised all of the tables and figures. Because we only discarded one genotype, these changes had no effect on result and conclusion.

**Comment**

The last point raised by the reviewer would be most important. Please carefully examine all the comments and revise the manuscript.

**Response**

We take the last point is the possibility of the misclassification of the synonyms. We added the probability of identity, that is quite low (6.56× 10^−35^). It is unlikely to detect false synonyms with the 31 SSRs. We would like to show you the number of markers which can distinguish in the combination of all the two varieties (N_of_markers_distinguish.xlsx). Except for synonyms, cultivars can be distinguished with small numbers of markers.

**Responses to the comments from Reviewer 3**

**Comment**

In cases of synonymous groups, authors omitted country name originated. Please use same notation.

199 Yanchang Beijing (China) C_OR (12)

Yanhong Beijing “no description of country here” C_OR (12)

**Response**

In response to this suggestion, we add the country names.

**Comment**

Use alphabetical numeral letters in a case of less than 10.

253 we were able to infer both parents, 5 cultivars were F1 hybrids between Japanese and Chinese

254 chestnut and 1 cultivar, ‘Ikaba’ (ID#67), was presumed to be an offspring between Japanese–

353 18 Japanese–Chinese cultivars, we were able to presume both parents for 6 cultivars and one

354 parent for 10 cultivars (Table 4).

**Response**

In response to this suggestion, we revised the sentences.

**Comment**

Is there a chance authors to misclassify synonymous or different types based on restricted number of SSRs. Because no other detail characteristics you can add to this data. Only a single evidence is not enough. You should refer the possible case in discussion section.

Authors mentioned Japanese case in the following reference but they are restricted genetic resources. In china, probably more numbers of resources and there might be similar but not the same landraces. At least need to reveal the possibility.

**Response**

We do not think there are misclassification of synonymous based on the 31 SSRs. We added the probability of identity, that is quite low (6.56× 10^−35^). It is unlikely to detect false synonyms with the 31 SSRs.

For fruit and nut species, it is typical that cultivars were spread by clonal propagation and the new names were given by local breeders and farmers. Because the cultivars in tree species have high heterozygosity and number of alleles in each locus are high, it is not difficult to distinguish cultivars using small numbers of SSRs. In most cases, the number of the SSRs to identify the synonyms were less than ours.
